# Blood transfusion for children in sub-Saharan Africa: 200 years on

**DOI:** 10.1016/S2352-4642(23)00143-8

**Published:** 2023-06-14

**Authors:** Sophie Uyoga, Dora Mbanya, Elizabeth C George, Kathryn Maitland

**Affiliations:** Kenya Medical Research Institute (KEMRI)–Wellcome Trust Research Programme, Kilifi 80108, Kenya; Faculty of Medicine and Biomedical Sciences, University of Yaoundé I, Yaoundé, Cameroon; Medical Research Council Clinical Trials Unit, University College London, London, UK; Department of Infectious Disease, Imperial College, London, UK; Institute of Global Health and Innovation, Faculty of Medicine, Imperial College, London, UK

In 1818, James Blundell completed the first successful human blood transfusion. This life-saving medical intervention has revolutionised patient care globally in the past two centuries. Nevertheless, many challenges remain in implementing safe blood transfusion procedures, particularly in sub-Saharan Africa^1^ where demand predominantly centres on acute paediatric severe anaemia and haemorrhagic complications of pregnancy and trauma. Severe anaemia, when the haemoglobin concentration drops below 6 g/dL, is very common in children admitted to hospital in sub-Saharan Africa. Infectious diseases, nutritional deficiencies, and sickle cell anaemia are the major underlying causes of anaemia. Outcomes remain unsatisfactory, with high mortality and high hospital readmission rates within 6 months of initial admission.^2^ World Blood Donor Day on June 14, 2023, calls for donors to “Give blood, give plasma, share life, share often”. Not only is this a day to celebrate the charitable contributions of blood donors, it is an important opportunity to raise awareness that every pint of blood donated results in critically important life-sustaining therapy, especially for children in sub-Saharan Africa.

In 1975, WHO recommended that countries promote national blood transfusion services based on voluntary non-remunerated donations and that they promulgate laws to govern their operation. Nevertheless, nearly 50 years later, issues around blood safety, adequate supply, equitable access, and rational use are still major challenges in many low-income countries. In 2004, WHO estimated that blood requirements in African countries were approximately 10–20 units donated per 1000 population per year.^3^ The actual blood requirements for each country’s population are not known, but a 2019 report^4^ suggests that many sub-Saharan Africa countries still collect less than 5 units per 1000 population annually. Estimates of the blood demand-to-supply ratio at a national level showed the magnitude of the shortage of blood for transfusion in this part of Africa.^4^ Although the situation is improving, progress is much too slow. Blood donation is not part of the culture in sub-Saharan Africa. Supply is further hampered by the many myths and taboos that are associated with blood donation in the region, and these are challenging to address.

**Figure:**
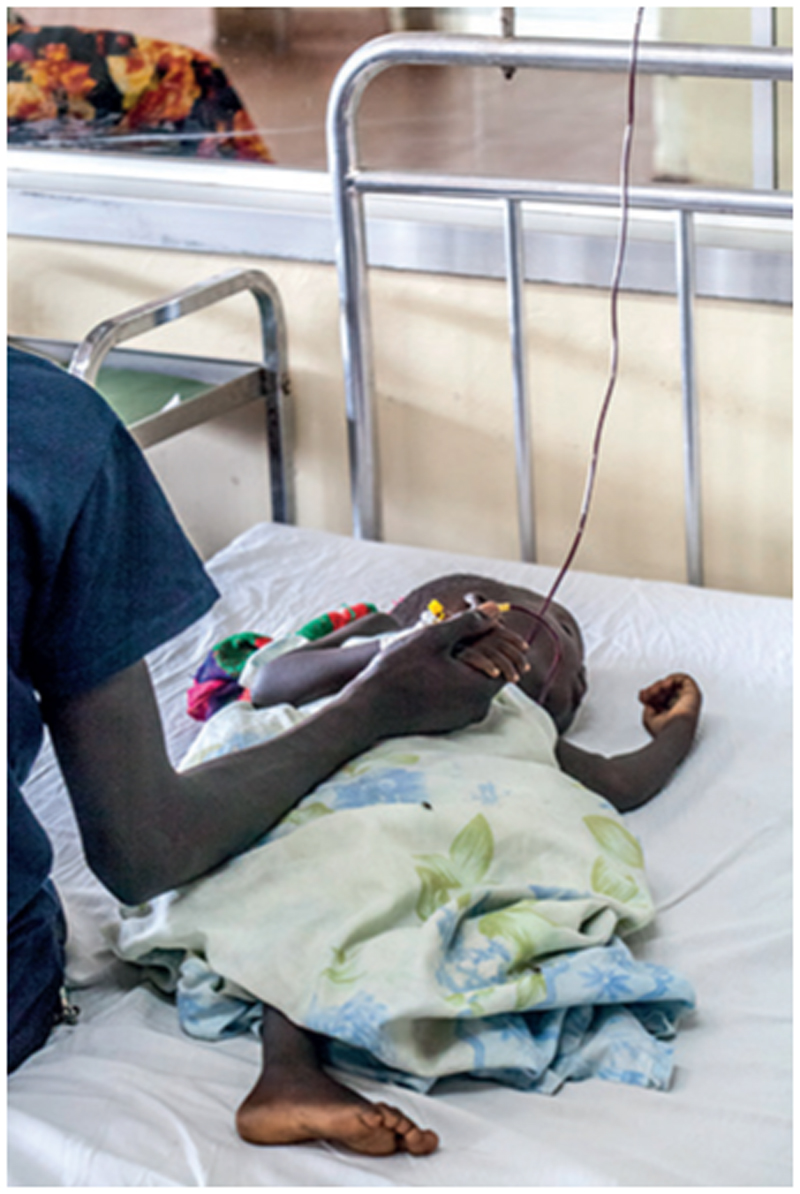
Mauro Fermariello/Science Photo Library

Uncertainty remains in relation to national blood collection and requirement estimates in sub-Saharan Africa, but what is certain is that children suffer the greatest impact of blood shortages.^1^ Most cases of acute paediatric severe anaemia present to hospitals as emergencies and are seasonally affected. Demands for blood are, therefore, highly unpredictable and challenging for blood transfusion service planning.

To protect scarce blood resources, rational use of blood has been recommended for children with severe anaemia.^5^ In the Transfusion and Treatment of severe Anaemia in African Children Trial (TRACT),^6^ we investigated two transfusion strategies in 3983 Malawian and Ugandan children aged 2 months to 12 years to improve the evidence base for the WHO guidelines that recommend against performing transfusions in African children hospitalised for uncomplicated severe anaemia. We found that for children with uncomplicated anaemia (haemoglobin 4–6 g/dL), no immediate transfusion was safe^6^ provided that the children were monitored for clinical signs or symptoms of severity and that haemoglobin was monitored to identify those developing profound anaemia (haemoglobin <4 g/dL) after admission. TRACT also provided strong evidence for improved outcomes for volume of blood transfusions that depended on fever status (ie, axillary temperature above or below 37·5°C). In 2018 and 2020, these trial findings were discussed at stakeholder meetings attended by paediatricians from ten countries in sub-Saharan Africa who are involved in national training and guideline development and in blood transfusion services. A new transfusion algorithm was developed to support implementation of these findings,^7^ and advocacy for point-of-care haemoglobin testing is ongoing.^8^

To align with international recommendations, and to maximise the use of a single blood donation, exclusive component preparation is now recommended across Africa. Many countries have struggled to implement exclusive component preparation, and the evidence supporting the superiority of red cell components (packed cells) over whole blood in the most common recipients throughout much of sub-Saharan Africa is weak.^9^ Transfusions are largely for emergency management, when the deficit to be replaced is whole blood. A secondary analysis of TRACT showed that whole blood was safe to use in children and resulted in superior haematological correction, reduced repeat transfusion, and shorter hospital stays.^10^ These findings have substantial cost implications for both blood transfusion and health services.

Collaborative work such as TRACT is greatly needed, with involvement of all stakeholders to identify mechanisms to improve implementation and safety of blood transfusion. Data from the African continent are insufficient. More evidence is needed to ensure good use of blood products. The high prevalence of transfusion-transmissible infections, and the general inaccessibility of appropriate screening techniques (eg, pathogen reduction and inactivation, and nucleic acid testing) all hamper safety and availability of blood. Further research gaps waiting to be filled include in-depth cost economics of blood transfusion and component preparation, and the development of alloimmunisation in frequently transfused patients. Advocacy needs investment, and communities in sub-Saharan Africa can be educated on the importance of blood donation to address the deficit in blood and blood products. Targeted education and sensitisation of populations is indispensable but must be done using culturally sensitive tools and examples. Populations served by transfusion services can help resolve the deficit by becoming blood donors themselves.

As we celebrate World Blood Donor Day, we salute all donors who, by giving blood, share life itself, and we call for greater collaboration and stakeholder involvement among all involved in blood donation and blood transfusion research in sub-Saharan Africa.
